# The Function and Mechanism of OXCT1 in Tumor Progression as a Critical Ketone Body Metabolic Enzyme

**DOI:** 10.3390/biom16010004

**Published:** 2025-12-19

**Authors:** Wen Qin, Wenwei Hu, Yiting Geng

**Affiliations:** 1 Department of Oncology, The Third Affiliated Hospital of Soochow University, 185 Juqian Street, Changzhou 213003, China; 20245235107@stu.suda.edu.cn; 2 Jiangsu Engineering Research Center for Tumor Immunotherapy, The Third Affiliated Hospital of Soochow University, Changzhou 213003, China

**Keywords:** OXCT1, tumor, metabolic reprogramming, drug resistance, ketone body metabolism

## Abstract

3-Ketoacid CoA-transferase 1 (OXCT1) is a homodimeric mitochondrial matrix enzyme essential for ketone body metabolism. It catalyzes the reversible transfer of coenzyme A from succinyl-CoA to acetoacetate, playing a central role in extrahepatic ketone body catabolism. Accumulating evidence indicates that OXCT1 is dysregulated in various cancers, where it functions as an oncogene, driving tumor progression by modulating proliferation, metastasis, apoptosis, autophagy, and drug resistance. Its overexpression is associated with aggressive tumor behavior, metabolic adaptation, and poor clinical outcomes. This review systematically summarizes the molecular structure, biological functions, and regulatory mechanisms of OXCT1, highlighting its multifaceted roles in tumorigenesis and progression. Furthermore, we discuss its potential as a diagnostic biomarker, prognostic indicator, and therapeutic target, providing novel insights for developing OXCT1-based anticancer strategies.

## 1. Introduction

In recent years, global cancer incidence and mortality rates continue to rise, emerging as a critical public health challenge. According to 2022 statistics released by the World Health Organization (WHO) and the International Agency for Research on Cancer (IARC), approximately 20 million new cancer cases were diagnosed worldwide, with 9.7 million cancer-related deaths recorded in that year [1]. As the second leading cause of mortality globally, malignant neoplasms now represent one of the most pressing threats to human health in the 21st century.

Metabolic reprogramming is one of the central features of tumorigenesis, progression and therapeutic resistance in cancer. Beyond the classical Warburg effect, emerging evidence demonstrates that reactivation of unconventional metabolic pathways including lipid metabolism, amino acid metabolism and ketone body metabolism plays pivotal roles in supporting malignant progression. Ketone body metabolism, an adaptive metabolic pathway activated during fasting, ketogenic diets or diabetic conditions, provide an alternative energy source by oxidizing fatty acids to produce ketone bodies [2]. Ketone bodies mainly include acetoacetate (AcAc), 3-hydroxybutyrate (β-HB) and acetone, which are synthesized mainly in liver mitochondria. When the body is starved or in a low-carbohydrate state, fatty acids released from adipose tissue enter the liver, where they are oxidized by β-oxidation to produce large amounts of acetyl-coenzyme A (Acetyl-CoA), which is subsequently converted to ketone bodies by an enzymatic reaction. These ketone bodies are transported to peripheral tissues such as the brain, heart, and skeletal muscle, where they are reconverted to acetyl-CoA and enter the tricarboxylic acid cycle (TCA cycle), which provides energy for cellular life activities. This process plays a critical role in maintaining energy homeostasis and reducing protein hydrolysis during glucose deficiency. Ketone body metabolism plays a complex role in tumor progression. While tumor cells usually rely on aerobic glycolysis of glucose for energy, most cannot efficiently utilize ketone bodies due to deficiencies in key mitochondrial enzymes [3,4,5,6,7]. However, under glucose-deprived stress conditions, a subset of tumor cells can activate ketone body oxidation pathways, exploiting them as an alternative energy source to support survival and proliferation. Furthermore, ketone bodies may indirectly promote tumor progression not only by remodeling the tumor microenvironment through effects on oxidative stress and inflammation, but also by modulating relevant signaling pathways, such as those regulated by histone modifications [8].

The past decade has witnessed growing recognition of 3-oxoacid CoA-transferase 1 (OXCT1), a pivotal enzyme in ketone body metabolism, as a key regulator of metabolic adaptation in tumor cells. (This enzyme is encoded by the *OXCT1* gene. For clarity and consistency throughout this review, the italicized form *OXCT1* refers to the gene, while the standard form OXCT1 refers to its protein product.) Studies have shown that OXCT1 not only plays an important role in neurological disorders [9,10], skeletal-muscular disorders [11], cardiovascular disorders [12,13], and endocrine metabolism disorders [14,15], but also plays key roles in a variety of pathophysiological processes of tumors, such as cell growth and migration, mitochondrial proliferation, cellular ATP production, cancer cell autophagy and apoptosis, etc. [16,17,18] *OXCT1* demonstrates significantly differential expression patterns between tumor tissues and adjacent normal tissues across multiple cancer types, including hepatocellular carcinoma (HCC), colorectal cancer (CRC), lung cancer, bladder cancer (BCa), osteosarcoma (OS), ovarian cancer, prostate cancer (PCa), chondrosarcoma (CS), glioma, gastric cancer (GC), pancreatic cancer, nasopharyngeal carcinoma (NPC) and so on. In most cases, OXCT1 is overexpressed in tumor tissues, where it promotes cancer cell growth, proliferation, metastasis, and mediates drug resistance, ultimately leading to poor patient prognosis. Its encoded protein, OXCT1, emerges as a candidate biomarker for both cancer detection and outcome prediction [16]. Recent studies suggest that targeted inhibition of OXCT1-mediated ketone body metabolism may effectively disrupt tumor cell energy compensation pathways, potentially representing a breakthrough strategy for metabolic intervention in cancer [19]. This review focuses on elucidating the tumor-promoting roles and underlying mechanisms of OXCT1 as the central enzyme in ketone body metabolism, while evaluating its potential clinical applications as a predictive biomarker for cancer diagnosis, therapeutic decision-making, and prognosis assessment.

## 2. Synthesis, Structure and Biological Function of OXCT1

The *OXCT1* gene is located on human chromosome 5p13.1, spanning over 100 kb with 17 exons, and belongs to the 3-oxoacid CoA-transferase gene family [20]. Its 361 bp proximal region regulates basal promoter activity and contains two GC boxes, each of which binds to the ubiquitously expressed transcription factor Sp1 in vitro [21]. OXCT1 is a homodimeric mitochondrial stromal enzyme containing two active sites encoded by the *OXCT1* gene. Its protein structure consists of 520 amino acids with a molecular weight of approximately 56 kDa, and it belongs to the family of succinyl-coenzyme A:3-ketoacid coenzyme A transferase enzymes, also known as succinyl-CoA:3-ketoacid CoA Transferase (SCOT). This enzyme plays a central role in the catabolism of ketone bodies in extrahepatic tissues by catalyzing the reversible transfer reaction of coenzyme A from succinyl-coenzyme A to acetoacetate. Inherited SCOT deficiency [22,23] or mutations in the *OXCT1* gene [24,25] may result in life-threatening recurrent ketoacidotic episodes.

The OXCT1 amino acid sequence contains key active sites: a glutamate residue, which is responsible for catalysis, and the substrate-binding region, which contains specific structural domains for the binding of 3-ketoacids (e.g., acetoacetate) and coenzyme A (CoA). The glutamate residue is central to the catalytic reaction, while the specific structural domain ensures proper orientation of the substrate in the catalytic center. In the catalytic process, OXCT1 facilitates the transfer of a CoA moiety from succinyl-CoA to acetoacetate, producing acetoacetyl-CoA and succinate. The resulting acetoacetyl-CoA is then converted into acetyl-CoA by thiolase, which subsequently enters the tricarboxylic acid (TCA) cycle to provide energy. The N-terminus of OXCT1 contains mitochondria-targeting signaling sequences, which allows OXCT1 to be localized mainly in the mitochondrial matrix and to become a key participant in mitochondrial energy metabolism.

OXCT1 is usually found in dimeric form, where the two monomers are linked by a non-covalent bond to maintain the protein’s conformation and function. Although each monomer is independently involved in catalytic reactions, the dimer structure is critical for its stability and function. Under normal physiological conditions, OXCT1 is highly expressed in high-energy-demanding tissues (e.g., heart, kidney, skeletal muscle, and brain), whereas it is barely expressed or suppressed in the liver [26], due to the fact that the liver is mainly responsible for the synthesis of ketone bodies rather than their utilization to avoid futile recycling. It has been suggested that the liver-specific silencing of *OXCT1* gene expression may be partially mediated by its 2.2 kb flanking sequence at the 5′ end [21], and there is also evidence that this process may be associated with the hypermethylation status of the CpG islands in the promoter region [27]; however, this idea remains controversial, and it has been argued that the methylation status of the CpG islands in the promoter region is not involved in this regulatory process [28]. This tissue-specific expression distribution ensures the effectiveness of the ketone body metabolic process, which not only provides energy for the high-energy-consuming organs, but also avoids wasting ketone body resources by the liver itself, reflecting the precision and adaptability of the organism’s metabolism.

In addition, a human testis-specific succinyl-CoA:3-oxoacid CoA-transferase 2 (OXCT2, also known as h-Scot-t) exists, which is encoded by the *OXCT2* gene [29,30]. This gene is an intronless single-copy gene specifically expressed in testicular haploid spermatocytes, and the protein it encodes shares 74% amino acid sequence homology with OXCT1 [31]. The high degree of similarity between OXCT2 and OXCT1 suggests that it may be involved in the regulation of energy metabolism of the reproductive system through a functional compensatory mechanism under specific physiological conditions.

## 3. Regulation of OXCT1 Expression

**Figure 1:**
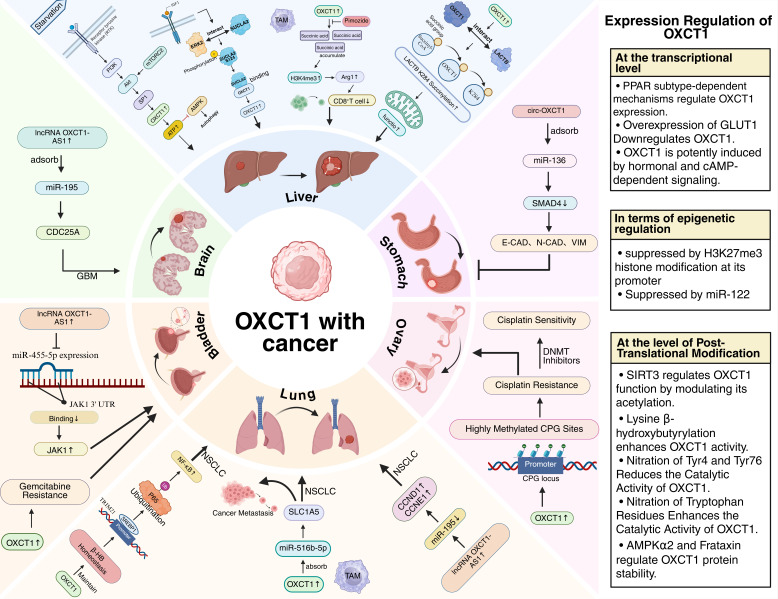
Representative mechanisms by which OXCT1 promotes tumor progression and its reg-ulatory pathways. The left panel summarizes the tumor-promoting functions of OXCT1 in repre-sentative cancers: liver (references [17,33,34,35]), Stomach [36], ovary [37], lung [38,39,40], bladder [41,42], and brain [43]. The right panel outlines the principal mechanisms regulating OXCT1 ex-pression, with comprehensive details provided in Section 3 “Regulation of OXCT1 expression”. (Arrows adjacent to related molecules indicate upregulation or downregulation. Created with Bi-oRender.com (accessed on 23 May 2025)).

OXCT1 activity is a major determinant of tissue ketolysis capacity, and its function may be regulated by a variety of factors to adapt to different metabolic states [8,32]. However, studies on the regulatory mechanisms of *OXCT1* gene and protein expression are still relatively limited at the cellular level. Primarily encompassing three mechanistic tiers: transcriptional control, epigenetic modification, and post-translational regulation, as shown in Figure 1 (right).

At the transcriptional level, *OXCT1* expression is precisely modulated by nutrient status and hormonal signaling: (1) PPARα is a fatty acid ligand-activated nuclear receptor transcription factor that promotes fatty acid utilization during hepatic and cardiac fasting. In the state of ketosis, the expression of *OXCT1* and the production of its encoded ketolytic enzyme OXCT1 are suppressed, whereas high uptake/supply of fatty acids or glucose also inhibits the expression of the *OXCT1* gene and thus ketolysis, which may be closely related to the regulation of PPAR subtype-dependent mechanisms [44]; (2) Cardiac-specific overexpression of the insulin-independent glucose transporter GLUT1 (Glucose Transporter Type 1) downregulates *OXCT1* expression by 67% in cardiomyocytes [45]; (3) In the brain, OXCT1 exhibits cell-type-specific expression and activity patterns, showing potent induction by both hormonal regulators (glucocorticoids and thyroid hormones) and cAMP-dependent signaling pathways [46,47].

In terms of epigenetic regulation, *OXCT1* expression in the liver is suppressed by promoter H3K27me3 histone modification (trimethylation of lysine 27 on histone H3) and liver-specific microRNA miR-122 [48], this phenomenon that is particularly pronounced during the transition from fetal to neonatal life.

OXCT1 activity is dynamically regulated at the level of Post-Translational Modification (PTM): (1) Acetylation modification: as a NAD^+^ dependent mitochondrial deacetylase, SIRT3 (Sirtuin 3) plays a key role in the function of OXCT1 by regulating its acetylation level [10,49]. In SIRT3-deficient mice, multiple lysine residues of OXCT1 (especially K451) are hyperacetylated, significantly reducing its catalytic activity, whereas SIRT3 restores the function of OXCT1 through deacetylation and ensures that ketone body metabolism proceeds normally; (2) β-Hydroxybutyrylation modification: β-HB dynamically regulates ketone body metabolic homeostasis by inducing lysine β-hydroxybutyrylation (Kbhb) at residue K421 of OXCT1, thereby enhancing its enzymatic activity and establishing a positive feedback regulatory loop [50]. (3) Non-enzymatic nitrification modification: in the heart of diabetic mouse model (db/db mice), nitrification targeted two key tyrosine sites of OXCT1 (Tyr4 and Tyr76), significantly reducing their catalytic activity, and the enzymatic activity of OXCT1 was maintained by protecting these two sites from nitration modification through targeted mutagenesis [51,52,53]. In addition, it has been shown that nitration of tryptophan residues instead enhances OXCT1 activity [54,55], further revealing the dual effects of nitration modification on OXCT1 function; (4) Protein stability regulation: AMP-activated protein kinase alpha 2 subunit (AMPKα2) [56] and Frataxin [57] independently stabilize OXCT1 by physically interacting with and inhibiting ubiquitin-proteasome-mediated degradation, thereby modulating systemic ketone body metabolism through enhanced OXCT1 protein stability. These regulatory mechanisms provide important clues for a deeper understanding of the role of OXCT1 in metabolic regulation, and also suggest that it may be a potential target for future metabolic disease intervention.

## 4. OXCT1 in Tumor Progression: Mechanisms and Implications

OXCT1 plays a complex role in tumorigenesis and progression through multiple mechanisms, and these mechanisms do not operate independently, but rather synergize and participate in concert with each other, ultimately affecting the biological behavior of tumors, as shown in Figure 1.

### 4.1. Provision of Energy Support and Regulation of Metabolic Reprogramming

Hypoxia and nutrient deprivation in the tumor microenvironment prompt tumor cells to activate multiple alternative energy acquisition pathways through metabolic reprogramming [58]. This metabolic flexibility involves not only the efficient utilization of conventional energy sources (e.g., glucose and glutamine), but also the ability to utilize non-conventional substrates, including lactate (which was historically regarded as a metabolic waste product), acetate, ketone bodies, and exogenous proteins [59]. In tumor cells, enhanced ketone body metabolism is considered an important adaptive strategy to maintain their energy supply in a hostile microenvironment. Previously, it was believed that OXCT1, a key enzyme in ketone body metabolism, was expressed at low levels in tumor tissues, especially in non-hepatic-derived tumors that may lack ketone body metabolizing capacity [60]. However, further studies have since revealed that OXCT1 is not only significantly expressed in HCC but also plays a crucial role in the efficient utilization of ketone bodies by a variety of malignant tumor cells.

Huang et al. [17] showed that in HCC cells, serum starvation conditions specifically activated *OXCT1* expression through activation of the mTORC2-AKT-SP1 signaling pathway, while inhibiting AMP- activated protein kinase (AMPK) activation and autophagy, which promoted the use of ketone bodies for ATP production and cell proliferation. Knockdown of *OXCT1* significantly inhibited the growth of HCC cells under serum starvation conditions, whereas restoration of *OXCT1* expression significantly restored intracellular ATP levels and growth capacity. Under stress conditions, HCC cells maintain survival and drive tumor progression by consuming ketone bodies, a convenient energy source. Guo et al. [33]’s study found that Insulin-like growth factor 1 (IGF1) induces extracellular signal-regulated kinase 2 (ERK2) to interact with succinate-CoA ligase ADP-forming subunit beta (SUCLA2) by binding to its receptor IGF1R, which makes the phosphorylation modification of serine 124 (S124) of SUCLA2. Phosphorylated SUCLA2 S124 promotes SUCLA2 binding to OXCT1 by recruiting PIN1 to cis-trans isomerize SUCLA2 through the P1N1 WW structural domain. SUCLA2 bound to OXCT1 produces succinyl coenzyme A, which on the one hand serves as a substrate for the OXCT1-catalyzed reaction; on the other hand, it directly promotes succinyl modification of the lysine at position 421 of OXCT1 (K421), which enhances the activity of OXCT1.The cascade of up-regulation of OXCT1 activity by SUCLA2 significantly increases AcAc-CoA, Acetyl-CoA and ATP production, which in turn promoted HCC cell proliferation and mouse tumor growth. Acetohydroxamic acid (AHA)–a clinically approved ureolytic inhibitor and small-molecule OXCT1 antagonist–demonstrates marked synergistic antitumor effects when combined with first-line agent lenvatinib in murine HCC models, establishing a novel translatable strategy for advanced hepatocellular carcinoma combination therapy.

The oncogene p53 exerts its function primarily at the transcriptional level. As a key transcription factor, p53 is mutated in more than 50% of human cancers, and p53-deficient mice exhibit significant cancer susceptibility [61,62]. In the metabolic regulation of CRC mutant p53 has been found to bind to promoter 1 within the 5′ upstream regulatory region of the *OXCT1* gene under the low-glucose stress induced by a Ketogenic Diet (KD), thereby upregulating OXCT1 expression [63]. This mutant p53-mediated metabolic reprogramming enables tumor cells to utilize ketone bodies as an alternative energy source, consequently leading to resistance to KD therapy. Based on the expression profiles of the metabolic enzymes OXCT1, ACAT1, GLUT1, and PFKFB3, Tang et al. [63] classified CRC into distinct metabolic subtypes. Among these, tumors characterized as glycolysis-proficient (G^+^, with overexpression of GLUT1/PFKFB3) and ketolysis-deficient (K-, with loss or low expression of OXCT1/ACAT1) are unable to efficiently utilize ketone bodies. As a result, their energy supply is severely compromised in the low-glucose environment created by KD. This metabolic vulnerability renders them sensitive to KD treatment, meaning their growth can be effectively suppressed. However, this sensitivity is not immutable in p53-mutant G^+^/K^-^ tumors. As noted, mutant p53 can induce OXCT1 expression, converting the tumor from a “ketolysis-deficient” to a “ketolysis-proficient” state, thereby conferring treatment resistance. Addressing this mechanism, research [63] demonstrates that combining KD with an allosteric activator of mutant p53, such as COTI-2, can block the upregulation of OXCT1 by mutant p53, suppress metabolic reprogramming, and consequently restore the tumor’s sensitivity to KD therapy.

### 4.2. Suppression of Antitumor Immunity by Epigenetic Modifications

Epigenetics refers to heritable changes in gene expression that do not involve alterations in the DNA sequence and are usually mediated by molecular modifications such as DNA methylation, histone modifications, and non-coding RNAs [64]. The liver, as a vital organ of the human body, is enriched with Kupffer cells and monocyte-derived macrophages. The development of programmed tumor-associated macrophages (TAM) in the HCC tumor microenvironment plays a critical role in tumorigenesis and progression. The high expression of OXCT1 in TAMs is closely associated with HCC progression. Analysis of clinical samples showed that TAM with high OXCT1 expression was significantly negatively associated with patient prognosis. Compared to normal macrophages, the high expression of OXCT1 in TAMs promotes the accumulation of the ketolytic metabolite succinate. Acting as a competitive antagonist of α-ketoglutarate, accumulated succinate inhibits the activity of histone lysine demethylases, particularly the KDM5 family members responsible for H3K4me3 demethylation [65,66]. This inhibition leads to an increase in H3K4me3 levels at the arginase 1 (Arg1) promoter, thereby upregulating Arg1 transcription. The upregulation of Arg1 weakens the cytotoxic effect of CD8^+^ T cells and induces their exhaustion [34]. These TAMs express PD-L1 and PD-L2, and through binding to CD8^+^ T cell surface receptors, they lead to functional exhaustion and cell death of CD8^+^ T cells, thereby promoting immune evasion and progression of HCC [67,68]. *OXCT1* conditional knockout mice showed significant inhibition of tumor progression in different hepatocellular carcinoma models. The OXCT1 inhibitor pimozide can alleviate CD8^+^ T cell exhaustion and inhibit TAM polarization toward a pro-tumor phenotype by downregulating the succinate-mediated H3K4me3-Arg1-CD8^+^ T cell axis, thereby suppressing HCC growth [34]. Given the negative regulatory role of OXCT1 overexpression in TAMs on HCC antitumor immunity, intervention targeting OXCT1 may be a potential strategy for the treatment of HCC.

### 4.3. Promotion of Post-Translational Modifications

Serine β-lactamase-like protein (LACTB) is a vertebrate-conserved mitochondrial intermembrane space protein that regulates mitochondrial lipid metabolism and other signaling pathways through its proteolytic activity, functioning as a tumor suppressor in various cancers including breast cancer, melanoma, and HCC [69,70]. Investigations from Ma’s group [35] revealed that OXCT1 is a newly discovered lysine succinyltransferase whose G424 site functions as an active center. Specifically, OXCT1 interacts directly with LACTB through its structural domain V and relies on structural domain IV to facilitate the transfer of the succinyl group from succinyl-CoA to OXCT1 and then to the K284 residue of LACTB, which leads to the succinylation of K284. This process inhibits the proteolytic activity of LACTB and enhances its metabolic function. Moreover, as a protease, LACTB downregulates the expression of mitochondrial phosphatidylserine decarboxylase (PISD), thereby reducing the mitochondrial levels of its metabolic products, lysophosphatidylethanolamines (LPE) and phosphatidylethanolamines (PE), consequently suppressing mitochondrial function [70]. In contrast, LACTB K284 succinylation not only enhances mitochondrial membrane potential, but also promotes mitochondrial respiration and function. Succinylation of LACTB K284 leads to the above-mentioned alterations in its function, which in turn promotes the genesis and progression of HCC cells. These findings further reveal a direct role for OXCT1 in protein modification metabolism, especially in the extensive promotion of lysine succinylation.

### 4.4. Activation of the NF-κB Signaling Pathway

Sterol regulatory element-binding protein 1 (SREBP1) is a key transcription factor in the basic helix-loop-helix leucine zipper (bHLH-Zip) family, which mainly regulates the expression of genes related to lipid metabolism [71]. Tripartite Motif-Containing Protein 21 (TRIM21) is an E3 ubiquitin ligase belonging to the TRIM family, which is involved in immunomodulation, antiviral defense and intracellular protein degradation [72]. Studies have shown that OXCT1 activates the NF-κB signaling pathway through the β-HB-SREBP1-TRIM21 axis, which promotes the development and progression of non-small cell lung cancer (NSCLC), and targeting OXCT1 can effectively inhibit tumor formation [38]. The specific mechanism is that OXCT1 maintains the homeostasis of β-HB, enabling it to function as a signaling metabolite [73] by acting through its cell surface receptor (e.g., GPR109A) [8] to inhibit the transcriptional activity of SREBP1. SREBP1 further binds to the promoter region of TRIM21, mediates ubiquitination of p65, and ultimately activates the NF-κB signaling pathway.

However, the regulation of the NF-κB signaling pathway by the ketogenic diet and β-HB appears to be highly variable. In multiple models of neurological injury and disease, both β-HB and the ketogenic diet have been demonstrated to suppress the NF-κB signaling pathway, thereby exerting potent anti-inflammatory effects. For instance, in models of spinal cord injury [74], ischemic brain injury [75], heat stress-induced neuroinflammation [76], and experimental autoimmune encephalomyelitis (EAE) for multiple sclerosis [77], ketogenic intervention or β-HB administration effectively inhibited NF-κB pathway activation through mechanisms such as suppressing the TLR4/NF-κB axis, upregulating IRAKM expression, or inhibiting the NF-κB/NLRP3 pathway. This functional paradox suggests that the net effect of β-HB is likely determined by cell type, disease context, or specific microenvironmental cues.

### 4.5. Mediation of Drug Resistance Development

Chemotherapy is one of the most important tools in tumor treatment, and its combination with surgery can significantly improve patient survival. Some studies have shown that drug-resistant in situ tumors exhibit OXCT1-dependent increases in mitochondrial oxygen consumption rate, ATP production and nucleotide biosynthesis. The mechanism of resistance to gemcitabine, a standard therapeutic agent for a variety of cancers including pancreatic, CRC, ovarian and BCa, is closely related to OXCT1 [78,79]. In drug-resistant BCa, overexpression of OXCT1 promotes gemcitabine resistance by driving back-complementation reactions, nucleotide biosynthesis, and dysregulation of OVOL1, and knockdown of *OXCT1* restores sensitivity of tumor cells to gemcitabine [41]. OVOL1, as a transcriptional repressor, is often thought to inhibit stem cell characteristics and epithelial–mesenchymal transition (EMT) and is a key regulator of gemcitabine resistance. Gemcitabine treatment reduces the intranuclear availability of OVOL1 by upregulating *OXCT1*, leading to cellular dedifferentiation and possibly inducing the expression of stem cell features associated with drug resistance. In addition, it was found that the vast majority of patients with pancreatic ductal adenocarcinoma (PDAC) develop resistance after gemcitabine treatment. The underlying mechanism is primarily associated with OXCT1 activating the NF-κB signaling pathway: upregulation of OXCT1 promotes the phosphorylation of IKKβ, IκBα, and P65, and drives the nuclear translocation of phosphorylated P65 (p-P65). This subsequently activates downstream anti-apoptotic genes, ultimately leading to gemcitabine resistance [80]. Therefore, targeting the NF-κB signaling pathway may represent a potential therapeutic strategy to suppress OXCT1-mediated gemcitabine resistance in PDAC, offering a new therapeutic option for patients with high OXCT1 expression.

The gradual development of resistance to cisplatin, a platinum-based first-line chemotherapeutic agent for ovarian cancer, is a major cause of disease recurrence. It was found that compared with the cisplatin-sensitive group, OXCT1 expression was significantly downregulated in the cisplatin-resistant group. This downregulation was associated with a highly methylated state at specific CpG sites in its promoter region, notably at positions −85, −70, and −66 upstream of the transcription start site (TSS), indicating epigenetic regulation of its expression. Further experiments demonstrated that treatment with a DNA methyltransferase (DNMT) inhibitor effectively reversed the hypermethylation of the *OXCT1* promoter and alleviated its mediated gene silencing, thereby restoring the chemosensitivity of ovarian cancer cells to cisplatin [37]. These findings establish OXCT1 as an epigenetically regulated resistance suppressor that critically influences cisplatin sensitivity in ovarian cancer. However, its specific downstream effectors and pathway mechanisms remain unclear, and elucidating this gap constitutes a priority for future research. Consequently, reversing its methylation status or restoring its expression represents a promising new strategy to overcome cisplatin resistance in recurrent ovarian cancer.

### 4.6. Regulation of Tumor Progression Through Non-Coding RNA Forms

In addition to encoding OXCT1 proteins with metabolic regulatory functions, the *OXCT1* gene can also generate non-coding RNA products with independent biological functions through different splicing methods, including circular RNAs (circRNAs) and long non-coding RNAs (lncRNAs). These non-coding RNA products are mainly involved in tumorigenesis and development through the ceRNA mechanism. Together, they constitute a multi-level regulatory network of the *OXCT1* gene locus, providing a new dimension for understanding its non-metabolic functions in cancer.

#### 4.6.1. CircRNAs

CircRNA 3-oxoacid CoA-transferase 1 (circ-OXCT1) is a functionally defined circRNA molecule formed by cyclization of exons 8 to 13 of the *OXCT1* gene, which plays the role of competitive endogenous RNA (ceRNA) by adsorption of microRNA (miRNA) and regulates the expression of downstream target gene mRNAs, thus participating in the regulation of various biological processes such as cell growth, metastasis, and apoptosis [81,82,83,84], which maintains the same basic characteristics as circ-RNA. CircRNAs are a class of non-coding RNAs formed by reverse splicing of linear Pre-mRNAs molecules [85], which are widely expressed in a variety of species and have high sequence conservation [86]. Compared to linear RNAs, circRNAs exhibit greater stability due to their closed-loop structure that is resistant to degradation by RNase R [87]. Studies have shown that CircRNAs exhibit dysregulated expression in various cancers and exert dual regulatory roles in both promoting and suppressing tumorigenesis [85,88,89,90,91]. Circ-OXCT1 regulates malignant behaviors and metabolism of tumor cells through different molecular mechanisms in NSCLC and GC.

In NSCLC, circ-OXCT1 acts as a molecular sponge by adsorbing miR-516b-5p, which in turn activates the miR-516b-5p/SLC1A5 signaling pathway and promotes proliferation, metastasis and glutamine metabolism in NSCLC cells [39]. Functional experiments showed that overexpression of miR-516b-5p significantly inhibited the malignant behavior of NSCLC cells, while silencing of miR-516b-5p partially reversed the inhibitory effect of circ-OXCT1 knockdown on the malignant behavior of NSCLC cells. Further mechanistic studies revealed that solute carrier family 1 member 5 (SLC1A5) was a direct target of miR-516b-5p. miR-516b-5p induced apoptosis of NSCLC cells by targeting and inhibiting the expression of SLC1A5, whereas overexpression of SLC1A5 could almost completely block the inhibitory effect of miR-516b-5p on the malignant phenotype of NSCLC cells. In addition, circ-OXCT1 also regulates GC progression through the circ-OXCT1/miR-136/SMAD4 signaling axis [36], and circ-OXCT1 binds and adsorbs miR-136 through its coding region (CDS) to inhibit the expression of SMAD4, which in turn regulates the expression of EMT-associated markers (including E-CAD, N-CAD and VIM) in GC cells, ultimately inhibiting EMT process and metastasis of GC cells.

#### 4.6.2. lncRNAs

lncRNAs are strings of non-coding transcripts more than 200 nucleotides in length [92], and a large number of lncRNAs are aberrantly expressed in tumor [93]. Although they have been regarded as “junk” or “transcriptional noise” in the genome [94], there is increasing evidence that they play a key role in the regulation of cell proliferation, invasion, apoptosis and other biological processes [95]. lncRNAs function as miRNA molecular sponges [96], by competitively binding to miRNAs, thereby inhibiting their regulation of downstream target gene expression [97].

The lncRNA/miRNA/target gene regulatory network was found to have important functions in a variety of tumors. For example, in NSCLC, the antisense RNA1 of lncRNA OXCT1 (lncRNA OXCT1-AS1) was highly expressed, while miR-195 was significantly under-expressed [40]. Knockdown of OXCT1-AS1 significantly up-regulated the expression of miR-195, inhibited the proliferation and colony forming ability of cancer cells, and down-regulated the expression of cell cycle regulators CCND1 and CCNE1. Further studies revealed that miR-195 could directly target the 3′ untranslated region (3′UTR) of CCNE1, whereas overexpression of CCNE1 reversed the effect of OXCT1-AS1 knockdown on NSCLC cells, suggesting that lncRNA OXCT1-AS1 promotes the proliferation of NSCLC cells by regulating the miR-195/CCNE1 axis. In addition, lncRNA OXCT1-AS1 stabilizes Lymphoid enhancer factor 1 (LEF1) by blocking E3 ubiquitin ligase NARF-mediated ubiquitination and enhances NSCLC metastasis [98]. In glioblastoma (GBM), lncRNA OXCT1-AS1 was significantly upregulated in both GBM patient tissues and cell lines. Functional experiments showed that inhibition of OXCT1-AS1 expression significantly reduced the proliferative capacity of GBM cells, induced cell cycle arrest, inhibited cell migration and invasion, and down-regulated the expression of EMT-related genes. lncRNA OXCT1-AS1 promotes GBM tumorigenesis by acting as a ceRNA for miR-195 and regulating the miR-195/CDC25A axis [43]. In BCa, OXCT1-AS1 promotes BCa cell proliferation and invasion by inhibiting the expression of miR-455-5p and decreasing its binding to the 3′ untranslated region of JAK1, thereby up-regulating JAK1 expression at the protein level [42]. In OS, lncRNA OXCT1-AS1 directly interacts with miR-886 and inhibits its maturation to promote OS cell proliferation [99].

In summary, existing research collectively establishes that lncRNA OXCT1-AS1 promotes tumorigenesis by sequestering specific miRNAs and regulating downstream oncogenic pathways, a mechanism that is independent of OXCT1’s ketolytic function. A critical question remains: despite its genomic overlap with the OXCT1 locus, it is still undetermined whether and how OXCT1-AS1 expression regulates the functional OXCT1 protein and thereby influences tumor ketone body metabolism. Elucidating this potential bridge between non-coding RNA function and core metabolic enzyme activity is crucial for a complete understanding of the multifaceted role of the *OXCT1* gene in tumor progression.

## 5. OXCT1 Expression in Tumors and Its Clinical Significance

OXCT1 is aberrantly overexpressed in various types of cancer, and its high expression is closely associated with a poor prognosis (Table 1). The following section will elaborate on the expression and clinical significance of OXCT1 in several major cancers.

### 5.1. OXCT1 and Hepatocellular Carcinoma

OXCT1 is significantly upregulated in HCC patients and can be used as an independent predictor of prognosis in HCC patients. Huang et al. [17] found that mRNA and protein levels of OXCT1 were significantly elevated in 20 HCC tissues as compared to normal tissues adjacent to the cancer. Immunohistochemical analysis of 158 HCC patients revealed significant enrichment of OXCT1 in malignant hepatocytes, and its expression level in advanced-stage (TNM III-IV) patients was notably higher than that in early-stage (TNM I-II) patients. In addition, OXCT1 expression level was positively correlated with tumor size and negatively correlated with patients’ survival time and vital status, etc. Combined TNM stage and tumor size could effectively predict the overall survival of HCC patients. Another study [35] found that the levels of OXCT1 and LACTB K284 succinylation in HCC tissues were significantly higher than those in normal tissues adjacent to the cancer, and were positively correlated with clinicopathological features. Notably, LACTB K284 succinylation levels were significantly accumulated in advanced HCC, and the survival time of patients with low expression was significantly better than that of patients with high expression and had a higher prognostic predictive value than that of total LACTB. The above findings were also confirmed by the study of Bai et al. [107]. In conclusion, OXCT1 can promote HCC progression through various mechanisms such as metabolic reprogramming, suppressing anti-tumor immunity and exerting succinyltransferase activity. These findings not only provide a new explanation for the role of OXCT1 in HCC, but also indicate that it is expected to be a reliable biomarker and a potential therapeutic target for the prognostic assessment of HCC.

### 5.2. OXCT1 and Colorectal Cancer

The expression level of OXCT1 in CRC has not been clearly defined, and there are inconsistencies in the reports of existing studies. By analyzing data from the Gene Expression Profiling Interactive Analysis (GEPIA) and The Cancer Genome Atlas (TCGA), Tang et al. [63] found that although the expression level of *OXCT1* gene varied in each CRC patient, its expression was generally decreased in CRC tissues compared with normal tissues adjacent to the cancer. Further analysis of transcriptome data from 782 patients showed that high expression of the ketolytic enzymes OXCT1 and ACAT1 was associated with a good prognosis in CRC patients, whereas high expression of the glycolytic enzymes GLUT1 and PFKFB3 suggested a poor prognosis. Subtype analysis based on metabolic profiling showed that patients with the G^+^/K^−^ subtype (glycolysis active/ketolysis inhibited) had the poorest prognosis (median survival 27 months), whereas patients with the G^−^/K^+^ subtype (glycolysis inhibited/ketolysis active) had the best prognosis (median survival > 60 months), and the mixed subtypes (G^−^/K^−^ or G^+^/K^+^) demonstrated intermediate survival, and patients with cancers of the G^+^/K^−^ subtype were the ideal population for KD. In the MAC16 mouse model of colon adenocarcinoma, OXCT1 showed low expression and activity, which may be related to the fact that tumor cells rely primarily on glucose metabolism and lack the ability to utilize ketone bodies [100]. However, Lee et al. [108] found that OXCT1 had a higher expression level in the metastatic CRC cell line CC-M3 by studying genes associated with CRC progression in feces, suggesting that it may serve as a potential marker for advanced CRC and can be detected in fecal samples, providing a new way to non-invasively study CRC occurrence and personalized prediction. The heterogeneity of these findings suggests that the expression pattern of OXCT1 in CRC may have significant individualized differences and tumor stage specificity, and its potential as a prognostic marker and therapeutic target still needs to be validated by further studies.

### 5.3. OXCT1 and Lung Cancer

OXCT1 showed significant upregulation of mRNA and protein levels in NSCLC, and its high expression was associated with poor patient prognosis. Knockdown of *OXCT1* significantly inhibited the growth and migration ability of NSCLC cells [38]. Circ-OXCT1 was also highly expressed in NSCLC tissues and cells, and regulated the proliferation, migration, invasion, apoptosis, and glutamine metabolism of NSCLC cells through the circ-OXCT1/miR-516b-5p/SLC1A5 signaling axis. In vivo experiments further confirmed that knockdown of circ-OXCT1 significantly inhibited tumor formation in mice in vivo, suggesting that it may serve as a potential biomarker and therapeutic target for NSCLC diagnosis and prognosis [39]. In addition, lncRNA OXCT1-AS1 was highly expressed in NSCLC cells and promoted tumor cell proliferation through the lncRNA OXCT1-AS1/miR-195/CCNE1 axis, and also stabilized the expression of LEF1 and enhanced the metastatic ability of tumor cells by blocking NARF-mediated ubiquitination. This further enriches the regulatory network of OXCT1-related molecules in NSCLC and provides new potential targets for precision therapy of NSCLC.

### 5.4. OXCT1 and Glioblastoma

Key enzymes of ketolysis, such as OXCT1, BDH1, and ACAT1, are significantly downregulated in both adult and pediatric GBM [101]. Patients with low expression of ketolytic enzymes in malignant gliomas, such as grade III interstitial astrocytomas and grade IV astrocytomas GBM, responded better to KD therapy than patients with positive expression [101]. Mechanistic studies have shown that OXCT1 promotes glycolysis by catalyzing ketolysis, which in turn promotes the proliferation of glioma cells, and may serve as a prognostic marker for glioma [102]. Based on these findings, targeting the GBM metabolic pathway through glycolysis inhibition in combination with a ketogenic diet or exogenous ketone body supplementation may be an effective adjunctive strategy to conventional therapy [109]. In addition, the expression levels of ketone body metabolism genes such as *OXCT1* can help screen brain tumor patients suitable for high-fat/low-carbohydrate KD therapy [5]. Conversely, the lncRNA OXCT1-AS1, encoded by the *OXCT1* gene, was significantly upregulated in GBM patients and tissue samples. Its high expression level was significantly associated with poor prognosis and reduced survival rates, particularly in patients with recurrent GBM [43]. OXCT1 and its related molecules play key roles in metabolic reprogramming and tumor progression in GBM, which not only provides new biomarkers for prognostic assessment of GBM, but also lays a theoretical foundation for the development of precision therapeutic strategies based on metabolic regulation, such as the ketogenic diet combined with targeted therapy. Future studies can further explore the specific molecular mechanisms of OXCT1 and its regulatory network in GBM to optimize the clinical application of metabolic intervention therapies such as ketogenic diet.

### 5.5. OXCT1 and Bladder Cancer

OXCT1 expression is up-regulated in BCa patients, and its high expression is significantly negatively correlated with shorter overall patient survival, progression of clinical stage, and poor efficacy of chemotherapy [41]. In addition, lncRNA OXCT1-AS1 was significantly overexpressed in BCa cell lines, promoting BCa cell proliferation and invasion by targeting miR-455-5p. Suggesting the potential role of OXCT1 and its related molecules as therapeutic targets for BCa [42].

### 5.6. OXCT1 and Prostate Cancer

OXCT1 as a key ketolysis enzyme is significantly upregulated in high-grade PCa (Gleason score > 8) and may serve as a potential tissue marker for the diagnosis and prognosis of high-grade PCa [103]. Labanca et al. [104] have also found that in both xenograft tumors and their matched human donor tissues, the expression of pivotal ketolytic enzymes—including ACAT1, OXCT1, and BDH1—was significantly enhanced following the progression to castration-resistant prostate cancer (CRPC). Elevated ACAT1 and OXCT1 expression correlated with increased risk of disease progression in PCa patients.

### 5.7. OXCT1 and Gastric Cancer

The study of OXCT1 in GC is gradually gaining attention. However, in addition to OXCT1 itself, its derived circular RNA molecule circ-OXCT1 also exhibits an important regulatory role in GC. Studies have shown that circ-OXCT1 expression is down-regulated in gastric cancer tissues and cell lines [36], and clinicopathological analyses of 74 pairs of gastric cancer and paracancerous tissues showed that circ-OXCT1 expression levels were significantly correlated with lymph node metastasis, pathological stage, and overall survival (*p* < 0.05), but no significant correlation was found with tumor size, gender, age, and clinicopathological features such as Borrmann’s classification. circ-OXCT1 low-expression patients had a significantly lower 5-year survival rate, suggesting that it may serve as a potential therapeutic target for advanced gastric cancer, especially for those with distant metastases.

### 5.8. OXCT1 and Breast Cancer

High expression of OXCT1 induces breast cancer cell growth and metastasis, and it may function as a metabolic oncogene in breast cancer progression [18]. However, OXCT1 expression in breast cancer is cell line-specific, and in the available studies, decreased OXCT1 expression did not significantly reduce survival of breast cancer patients [110], in contrast, high expression of BDH1 and ACAT1 was significantly associated with shorter patient survival. Breast cancer cells may rely on other metabolic pathways, such as glutamine metabolism, rather than ketone body metabolism to maintain energy supply under glucose deprivation conditions, suggesting that OXCT1 may not be a major driver of breast cancer cell metabolism. However, screening for expression levels of OXCT1 and other ketone body metabolizing enzymes may be helpful in assessing the suitability of patients for ketogenic diet therapy.

### 5.9. OXCT1 and Thymoma

OXCT1 is significantly aberrantly expressed in thymoma and may act as a driver gene to promote thymoma progression in synergy with other driver genes, such as FANCI, NCAPD3, NCAPG, EPHA1, and MCM2 [105]. KEGG pathway analyses suggest that OXCT1 may contribute to thymoma progression through mechanisms such as participating in cell cycle regulation, influencing the p53 signaling pathway, and mediating immunodeficiency-associated pathway, especially through synergistic effects with cell cycle-regulated genes such as MCM2.

### 5.10. OXCT1 and Pancreatic Ductal Adenocarcinoma

OXCT1 is highly expressed in PDAC, and its expression level is positively correlated with lymph node metastasis and vascular infiltration, and significantly negatively correlated with Recurrence-Free Survival (RFS) [80]. Patients with high expression of OXCT1 exhibited shorter RFS than patients with no or low expression of OXCT1, and one of the mechanisms is that OXCT1 promotes PDAC resistance to gemcitabine through the NF-κB signaling pathway, suggesting that targeting OXCT1 may be a potential therapeutic strategy for reversing PDAC gemcitabine resistance. However, whether OXCT1 is involved in PDAC progression and resistance through other molecular mechanisms still needs to be further explored.

### 5.11. OXCT1 and Osteosarcoma

OXCT1 expression levels correlate with ifosfamide (IFO) resistance in OS cells, where higher OXCT1 expression is observed in sensitive cell lines, while its expression becomes suppressed upon induced resistance and remains consistently low in resistant cell lines regardless of IFO treatment. Notably, these resistant cells regain IFO sensitivity when cultured under drug-free conditions, suggesting that OXCT1 expression dynamics may participate in regulating OS chemoresistance, although other signaling pathways or metabolic mechanisms might also contribute [111]. Furthermore, lncRNA OXCT1-AS1 is significantly upregulated in OS tissues and strongly associates with tumor size, where it promotes malignant phenotypes in OS cells [99]. The combined evaluation of OXCT1-AS1 overexpression and the mature/precursor miR-886 ratio in OS could potentially serve as a novel prognostic biomarker for patients.

## 6. OXCT1 and the Ketone Body Metabolism: Therapeutic Paradox and Prospects in Cancer

Under a normal dietary state, systemic ketone body levels remain typically low. This physiological context poses a critical question: under what physiological or pathological conditions does OXCT1, as the key enzyme for ketone body metabolism, demonstrate its significance in tumors? We posit that the pivotal role of OXCT1 becomes particularly salient during metabolic challenges, such as fasting, KD, or within the tumor microenvironment. The latter, through mechanisms of metabolic symbiosis, can create a local “niche” with elevated ketone body concentrations, thereby activating the ketolytic pathway.

Therefore, although the ketolytic function of OXCT1 is crucial under specific metabolic conditions, the KD, which acts by targeting its substrates (ketone bodies), exhibits significant and contradictory efficacy in oncology. On one hand, several studies support the tumor-suppressive role of KD [32,112]. For instance, in CRC, the ketone body β-HB acts through its surface receptor Hcar2, inducing the expression of the transcriptional regulator Hopx, which subsequently alters gene expression profiles and ultimately inhibits the proliferation of intestinal epithelial cells [112]. In some tumors with low expression of ketolytic enzymes, such as certain gliomas, the deficiency of key ketone-oxidizing enzymes like OXCT1 prevents cancer cells from efficiently utilizing ketone bodies as an alternative energy source, thereby predicting a better response to KD therapy [3,113]. Furthermore, KD has been demonstrated to enhance the efficacy of conventional radiotherapy and chemotherapy through mechanisms such as increasing oxidative stress and suppressing pro-inflammatory pathways [114,115]. On the other hand, however, evidence also indicates that KD may promote tumor progression under certain contexts. This tumor-promotive effect is predominantly observed in tumors with high OXCT1 expression or those possessing metabolic plasticity. These cells can efficiently convert circulating ketone bodies into energy, potentially even gaining a proliferative advantage. For example, in p53-mutant CRC, KD induced low glucose stress upregulates OXCT1 expression, conferring resistance to KD and enabling the utilization of ketone bodies to drive tumor growth [63]. Additionally, the high levels of circulating fatty acids and ketone bodies resulting from KD may influence tumor behavior via epigenetic mechanisms. Modifications such as histone β-hydroxybutyrylation can activate pro-survival signaling pathways [8,17,116,117]. In summary, KD demonstrates a dual role in cancer therapy, exhibiting both inhibitory and promotive effects. The underlying mechanisms for this paradox have not been fully elucidated. This contradiction suggests the potential involvement of yet unidentified metabolic regulatory pathways or microenvironmental factors in determining the ultimate biological impact of KD on tumors, warranting further in-depth investigation.

As a key ketolytic enzyme, OXCT1 drives ketolysis to supply energy for tumors and promotes tumor progression through various molecular mechanisms. Recent studies, however, have revealed that the role of OXCT1, which is central to understanding the paradoxical effects of ketone bodies in tumors, extends far beyond this. Historically, the role of ketone bodies in tumor biology has been primarily defined as that of an alternative energy source, where they act as energetic substrates (via OXCT1) to promote tumor development [8]. In recent years, however, research has uncovered two novel roles of ketone bodies, particularly β-HB, that go beyond their function as energy substrates: not only can it function as a molecule that indirectly modulates epigenetic states [118], such as by serving as an inhibitor of histone deacetylases (HDACs) [116], but it has also been identified as the direct donor constituting a novel type of histone modification [117]; meanwhile, it can also act as a signaling molecule that activates cell surface receptors to trigger pathways suppressing tumor proliferation [112].

This presents a paradox: how can the same molecule suppress tumors through signaling pathways yet promote tumors via catabolism? Considering the cellular function of OXCT1, we hypothesize that its expression level may serve as a core molecular switch determining the balance between these dual roles of ketone bodies. When OXCT1 expression is low in tumor cells, ketone bodies are not efficiently catabolized, allowing their signaling functions to dominate and thereby rendering KD more likely to exhibit tumor-suppressive effects. Conversely, in tumors with high OXCT1 expression, ketone bodies are effectively channeled into mitochondria for ATP production, favoring their role as an energy substrate over their signaling functions. This shift may explain why KD can be ineffective or even tumor-promotive in such contexts.

The operation of this OXCT1-mediated switch is critically dependent on intracellular ketone body availability, which is regulated upstream by specialized transmembrane transporters. Key influx transporters such as SLC5A8 [119] and MCT1 [120,121] facilitate ketone body uptake, while efflux mechanisms potentially involving other MCT isoforms [120] enable their export. Based on these functional relationships, we hypothesize that ketone body transporters and OXCT1 collectively form an integrated “gatekeeper-switch” axis that determines the metabolic fate of ketone bodies in tumors. This regulatory axis operates within the complex architecture of the tumor microenvironment, where it supports metabolic symbiosis between distinct cellular subpopulations. Drawing analogy to the established “lactate shuttle” model, we propose that certain cells, including specific cancer cells or cancer-associated fibroblasts [122,123], may produce and export ketone bodies, while adjacent cell subpopulations equipped with high expression of both ketone body transporters and OXCT1 efficiently import and oxidize these metabolites as fuel. In summary, we present a unified model in which the therapeutic efficacy of KD and the ultimate role of ketone bodies in tumors are determined not by any single molecular component, but by the functional configuration of the “ketone body transporter (gatekeeper)-OXCT1 (switch)” axis within the tumor ecosystem. Future research and therapeutic strategies should transcend the simplistic “ketone bodies: beneficial or harmful” dichotomy. Instead, they should target multiple facets simultaneously by inhibiting ketone body production and efflux, blocking their uptake, and disrupting OXCT1 function, thereby effectively intervening in this complex metabolic network and opening new avenues for precision tumor metabolism therapy.

## 7. Discussion

Despite significant advances in cancer diagnosis and treatment technologies in recent years, tumor prevention and management still face numerous challenges, including unclear etiology, undefined pathogenesis, lack of early diagnostic biomarkers, chemotherapy resistance, treatment-related toxicities, and difficulties in controlling recurrence and metastasis. Therefore, in-depth elucidation of tumorigenesis mechanisms, discovery of novel therapeutic targets, improvement of early diagnosis rates, and prognosis enhancement have become current research priorities.

Several studies have confirmed that OXCT1 plays an oncogene function in a variety of tumors and participates in tumor progression by promoting tumor cell growth, proliferation, invasion, metastasis and drug resistance, and has the potential to serve as a tumor diagnostic and prognostic marker. However, the following important scientific issues remain to be resolved in current research on OXCT1: first, although studies have revealed the prognostic value of OXCT1 in HCC, CRC, NSCLC and GBM, its prognostic significance in more tumor types has not yet been clarified, and the clinical sample sizes of some of the studies are relatively small, which needs to be systematically evaluated by expanding the sample sizes and tumor spectrum. Second, the study on the cancer-promoting mechanism of OXCT1 is still in the primary stage, although some of the molecular mechanisms have been preliminarily elucidated in HCC, whether these findings are tumor type-specific or not, and whether there are other undiscovered regulatory mechanisms need to be answered by more in-depth mechanistic studies. Third, the study of the association between OXCT1 and tumor drug resistance is still incomplete, the existing evidence only initially indicates that it is associated with drug resistance in BCa, PDAC and ovarian cancer, but the specific molecular mechanisms have not been elucidated, and the role of OXCT1 in drug resistance in other tumor types remains to be explored; fourth, the *OXCT1* gene forms a complex regulatory network through different transcripts, circ-OXCT1 and lncRNA OXCT1-AS1, although originating from the same gene locus, are regulated through a network of differentiated competitive endogenous RNAs (compliant endogenous RNAs), such as circ-OXCT1/miR-516b-5p/SLC1A5 [39], circ-OXCT1/miR-136/ SMAD4 [36], lncRNA OXCT1-AS1/miR-195/CCNE1 [40], lncRNA OXCT1-AS1/miR-195/CDC25A [43], lncRNA OXCT1-AS1/miR-455-5p/JAK1 [42], synergistically regulating tumor metabolic reprogramming and malignant progression. However, whether these non-coding RNAs regulate OXCT1 expression and function through specific pathways with feedback, or whether they regulate tumor growth, metabolism, and drug resistance through mechanisms other than ceRNAs remains unclear, and further mechanistic studies are needed to elucidate this complex regulatory network.

Tumorigenesis is a multifactorial process in which metabolic reprogramming plays a critical role. As the rate-limiting enzyme in ketone body metabolism, OXCT1 participates in metabolic reprogramming through multiple mechanisms and represents an important target for therapeutic intervention. However, KD, as one such interventional strategy, exhibits a paradoxical effect of both tumor suppression and promotion in cancer treatment. In light of this paradox, we hypothesize that OXCT1 and ketone body transporters collectively form a “gatekeeper–switch” axis. Within this axis, the expression level of OXCT1 serves as a key molecular switch that regulates the metabolic fate of ketone bodies, determining the balance between their tumor-suppressive signaling functions and tumor-promoting energetic roles. Future studies should focus on systematically validating this axis and elucidating how the tumor microenvironment precisely regulates OXCT1 expression. This research direction will provide a critical theoretical foundation for developing individualized therapeutic strategies targeting tumor metabolism.

Another key enzyme of ketolysis, ACAT1, similar to OXCT1, can also be involved in the progression of many tumors through mechanisms such as regulating antitumor immunity, modulating PTM, regulating metabolic reprogramming, and promoting the development of drug resistance [124,125], and correlating with prognosis. Some studies have shown that ACAT1 is an important new therapeutic target for further drug development and optimization [126]; for example, in HCC, ACAT1 can stabilize its function by acetylating the GNPAT K128 site, inhibit TRIM21-mediated ubiquitination degradation, and promote the growth of HCC cells [127]; ACAT1 expression is up-regulated in tumors such as CRC, lung adenocarcinoma, ER-negative breast cancer and so on, down-regulated in GBM, and when overexpressed in breast cancer, survival of breast cancer patients is reduced [125]. ACAT1 catalyzes the first step of ketone body production in the liver, and OXCT1 catalyzes the utilization of ketone bodies in extra-hepatic tissues, and the two work together to maintain the ketone energy supply and metabolic homeostasis, which suggests to us that OXCT1, as a key enzyme of ketolysis, may not play a role alone in tumor development, but may play a synergistic or antagonistic role with ACAT1 to promote tumor progression and affect tumor prognosis, so the study of the common mechanism of OXCT1 and ACAT1 is yet to be further carried out.

This study systematically reviews the research progress on OXCT1, encompassing its molecular structure and biological functions, expression regulation, key mechanisms in promoting tumor progression and drug resistance, as well as its expression patterns and clinical significance across cancers, while highlighting OXCT1’s dual value in oncology management: On the one hand serving as a potential biomarker for early detection and molecular classification in cancer diagnosis, on the other hand representing a promising therapeutic target for the development of OXCT1-targeted small-molecule inhibitors and prognostic evaluation in treatment strategies. However, OXCT1-mediated tumor progression is diverse and complex, and can act through the same mechanism in different tumors as well as through different mechanisms in the same tumor. In view of this, future research directions and challenges could focus on the following: (1) Delineate the upstream and downstream mechanisms of OXCT1 regulation. Expand research scope to investigate OXCT1’s functions and mechanisms across additional cancer types, establishing its metabolic regulatory network in tumors, while exploring the universality, heterogeneity, and interconnections of its oncogenic mechanisms; (2) Current OXCT1 studies predominantly rely on cell lines and xenograft models. Future work should employ more physiologically relevant models including tumor organoids and humanized mouse systems to better recapitulate OXCT1’s metabolic functions within authentic tumor microenvironments, thereby providing robust platforms for mechanistic exploration; (3) Investigate OXCT1’s role in remodeling tumor microenvironments, especially its interactions with immune cells and stromal cells; (4) Validate the correlation between OXCT1 expression patterns and tumor progression/patient outcomes through large-scale clinical cohorts, while rigorously evaluating its biomarker potential using standardized assays; (5) Conduct translational medicine research to develop OXCT1-specific inhibitors or modulators, and investigate their combined efficacy with existing therapeutic modalities including chemotherapy, radiotherapy, and immunotherapy, thereby providing novel strategies for personalized cancer treatment.

## 8. Conclusions

The importance of OXCT1 as a key enzyme of ketone body metabolism in tumor progression has been gradually highlighted, and it promotes tumor growth, proliferation, infiltration and metastasis, increases the malignant phenotype of tumors, and affects patients’ prognosis through a variety of mechanisms, such as metabolic reprogramming, inhibition of anti-tumor immunity, mediation of post-translational modifications, activation of the NF-κB signaling pathway, and inducing drug resistance. Although OXCT1 and its expression products have potential applications in tumor diagnosis, treatment and prognostic evaluation, their complex regulatory networks, in-depth mechanisms of cancer promotion and mediated drug resistance, as well as their prognostic and precision therapeutic value in multi-tumor populations remain to be further studied.

## Figures and Tables

**Table 1:** Expression, mechanism of action and relationship with prognosis of OXCT1 in different tumors.

Tumor Type	OXCT1 Expression Level	Patient Prognosis	Mechanism of Action	References
Hepatocellular carcinoma	high	Poor	Activation of the mTORC2-AKT-SP1 signaling pathway Role of the IGF1-OXCT1 metabolic axis Suppression of anti-tumor immune mechanisms Promotion of PTM	[17,33,34,35]
Colorectal cancer	indefinite	Poor	Regulation of metabolic reprogramming	[63,100]
Non-small cell lung cancer	high	Poor	Activation of NF-κB signaling pathway The circ-OXCT1/miR-516b-5p/SLC1A5 axis regulates cell function and metabolism The lncRNA OXCT1-AS1/miR-195/CCNE1 axis promotes cell proliferation lncRNA OXCT1-AS1 Stabilizes LEF1 by blocking NARF-mediated ubiquitination to enhance metastasis	[38,39,40,98]
Glioblastoma	low	Poor	LncRNA OXCT1-AS1/miR-195/CDC25A axis promotes tumorigenesis	[43,101,102]
Bladder cancer	high	-	Reduced intranuclear availability of OVOL1 The lncRNA OXCT1-AS1/miR-455-5p/JAK1 axis promotes cell proliferation and invasion	[41,42]
Prostate cancer	high	-	-	[103,104]
Lymphoma	high	-	-	[18]
Gastric cancer	low	Poor	Suppression of GC EMT and metastasis by attenuating the TGF-β pathway through the circ-OXCT1/miR-136/SMAD4 axis	[36]
Thymoma	abnormal	-	-	[105]
Osteosarcoma	abnormal	-	lncRNA OXCT1-AS1 interacts with miR-886 to promote OS cell proliferation	[99]
Pancreatic ductal adenocarcinoma	high	Poor	Promotion of PDAC resistance to gemcitabine through the NF-κB signaling pathway	[80]
Ovarian cancer	abnormal	-	Modulation of cisplatin sensitivity mediates changes in drug resistance	[37]
Nasopharyngeal carcinoma	high	Poor	-	[106]

Note: OXCT1 indicates 3-oxoacyl-coenzyme A-transferase 1, and—indicates that no relevant studies were retrieved.

## Data Availability

No new data were created or analyzed in this study. Data sharing is not applicable to this article.

## Abbreviations

The following abbreviations are used in this manuscript:

**Abbreviation**	**Definition**
OXCT1	3-Ketoacid CoA-transferase 1
WHO	World Health Organization
IARC	International Agency for Research on Cancer
AcAc	acetoacetate
β-HB	3-hydroxybutyrate
Acetyl-CoA	acetyl-coenzyme A
TCA cycle	tricarboxylic acid cycle
HCC	hepatocellular carcinoma
CRC	colorectal cancer
BCa	bladder cancer
OS	osteosarcoma
PCa	prostate cancer
CS	chondrosarcoma
GC	glioma, gastric cancer
NPC	nasopharyngeal carcinoma
SCOT	succinyl-CoA:3-ketoacid CoA Transferase
CoA	coenzyme A
OXCT2 or h-Scot-t	succinyl CoA:3-oxoaci ketoacid CoA-transferase 2
GLUT1	Glucose Transporter Type 1
H3K27me3	trimethylation of lysine 27 on histone H3
PTM	Post-Translational Modification
SIRT3	Sirtuin 3
Kbhb	lysine β-hydroxybutyrylation
AMPKα2	AMP-activated protein kinase alpha 2 subunit
AMPK	AMP-activated protein kinase
IGF1	Insulin-like growth factor 1
ERK2	extracellular signal-regulated kinase 2
SUCLA2	succinate-CoA ligase ADP-forming subunit beta
AHA	Acetohydroxamic acid
KD	ketogenic diet
TAM	tumor-associated macrophages
Arg1	arginase 1
LACTB	serine β-lactamase-like protein
PISD	phosphatidylserine decarboxylase
LPE	lysophosphatidylethanolamines
PE	phosphatidylethanolamines
SREBP1	sterol regulatory element-binding protein 1
bHLH-Zip	basic helix-loop-helix leucine zipper
NSCLC	non-small cell lung cancer
EMT	epithelial–mesenchymal transition
PDAC	pancreatic ductal adenocarcinoma
DNMT	DNA methyltransferase
circRNAs	circular RNAs
lncRNAs	long non-coding RNAs
circ-OXCT1	CircRNA 3-oxoacid CoA-transferase 1
ceRNA	competitive endogenous RNA
miRNA	microRNA
SLC1A5	solute carrier family 1 member 5
lncRNA OXCT1-AS1	antisense RNA1 of lncRNA OXCT1
LEF1	Lymphoid enhancer factor 1
GBM	glioblastoma
GEPIA	Gene Expression Profiling Interactive Analysis
TCGA	Cancer Genome Atlas
mOS	median Overall Survival
CRPC	castration-resistant prostate cancer
RFS	Recurrence-Free Survival
IFO	ifosfamide
HDACs	histone deacetylases
EAE	experimental autoimmune encephalomyelitis
